# Pertuzumab in HER2-positive breast cancer: a systematic review and meta-analysis of randomized controlled trials

**DOI:** 10.1007/s10549-026-08116-y

**Published:** 2026-11-09

**Authors:** Hala Aboushawareb, Abdelrhman W. Mohamed, Hamzah M. Alghzawi, Abdelrahman El Helbawy, Aanab Tarig, Huthayfa Zatari, Mohanad mahmoud Hekal, Islam Hasan, Ahmed Yasser Shaban

**Affiliations:** 1 Ministry of Health and Population, Cairo, Egypt; 2 Faculty of Medicine, Kafr Elsheikh University, Kafr Elsheikh, Egypt; 3 School of Nursing, Tennessee State University, Nashville, TN USA; 4 Nursing Department, Vanderbilt University Medical Center (VUMC), Nashville, TN USA; 5 Faculty of Medicine, Helwan University, Cairo, Egypt; 6 Faculty of Medicine, Ain Shams University, Cairo, Egypt; 7 Al-Quds University, Abu-Dies, Jerusalem, Palestine; 8 Faculty of Medicine, Alexandria University, Alexandria, Egypt; 9 Department of Internal Medicine, Holy Name Medical Center, Teaneck, NJ USA

**Keywords:** HER2-positive breast cancer, Pertuzumab, Trastuzumab, Dual HER2 blockade, Overall survival, Progression-free survival, Meta-analysis

## Abstract

**Background:**

HER2-positive breast cancer is a distinct subtype associated with less favorable outcomes without targeted therapy. Dual HER2 blockade with pertuzumab and trastuzumab has shown improved efficacy compared to single-agent approaches, but results across studies remain variable. This meta-analysis aims to evaluate the overall efficacy and safety of pertuzumab-based regimens.

**Methods:**

A systematic search of PubMed, Scopus, Web of Science, and the Cochrane Library was conducted to identify relevant RCTs. The quality of trials was assessed using the ROB-2 tool, and the certainty of the evidence was assessed using the GRADE tool. Data were synthesized using R.

**Results:**

This study included 8 RCTs with 8367 patients. Pertuzumab-based regimens were associated with improved overall survival. In the locally advanced or metastatic setting, a pronounced benefit was observed (HR = 0.71, 95% CI 0.65–0.77). However, in the neoadjuvant or adjuvant setting, the pooled hazard ratio (HR = 0.81, 95% CI 0.22–3.04), which was not significant. Progression-free survival (HR 0.74; 95% CI 0.67–0.83) was improved compared with the control group. In terms of safety, there was no significant difference in the incidence of any adverse events (risk ratio (RR) = 1.01; 95% CI 0.99–1.02) or grade ≥ 3 adverse events (RR 0.99; 95% CI 0.86–1.13) between the experimental and control groups.

**Conclusion:**

Pertuzumab-based dual HER2 blockade is associated with significant improvements in overall survival and progression-free survival in patients with metastatic HER2-positive breast cancer. Importantly, these survival benefits are not accompanied by a significant increase in overall or severe adverse events, supporting its favorable efficacy–safety profile across clinical settings.

**Supplementary Information:**

The online version contains supplementary material available at https://doi.org/10.1007/s10549-026-08116-y.

## Introduction

Breast cancer is one of the most frequently diagnosed malignancies among women worldwide [1]. Each year, approximately 2.3 million new cases are reported globally [1, 2]. Human epidermal growth factor receptor 2 (HER2)–positive breast cancer represents about 15–20% of all cases and is characterized by aggressive tumor behavior and poorer outcomes if not treated promptly [3]. Consequently, significant research efforts have focused on improving the diagnosis and management of this subtype, with anti-HER2 targeted therapies becoming a cornerstone of treatment [4]. Trastuzumab, a monoclonal antibody, has long been established as a key component of therapy in combination with chemotherapy for both early-stage and metastatic HER2-positive breast cancer. However, a substantial proportion of patients either fail to respond or eventually develop disease recurrence [5].

Pertuzumab is another HER2-targeted monoclonal antibody that inhibits HER2 dimerization with other HER family receptors, thereby blocking downstream signaling pathways involving HER2/EGFR and HER2/HER3 heterodimers [6]. Because pertuzumab and trastuzumab bind to distinct domains of the HER2 receptor, their combined use results in complementary antitumor effects, including enhanced inhibition of signaling and increased antibody-dependent cellular cytotoxicity [7]. Clinical trials have demonstrated that dual HER2 blockade with trastuzumab and pertuzumab is more effective than trastuzumab alone in treating early, locally advanced, and metastatic HER2-positive breast cancer [7]. Specifically, in the landmark phase III CLEOPATRA trial, adding pertuzumab to trastuzumab and docetaxel extended median overall survival to an unprecedented 57.1 months (HR 0.69, 95% CI 0.58–0.82) with a favorable long-term cardiac safety profile [8]. For the neoadjuvant setting, the NeoSphere study showed that adding pertuzumab to trastuzumab plus docetaxel improves pCR (pathological complete response) and is consistent with better 5-year outcomes, supporting pertuzumab-based neoadjuvant therapy [9]. Similarly, the phase III PEONY trial demonstrated the efficacy of this combination therapy strategy in Asian patients with either early or locally advanced HER2-positive breast cancer. Significant increases in both total pathologic complete response (tpCR) and 5-year event-free survival rate (84.8% vs. 73.7%; HR 0.53, 95% CI 0.32–0.89) were observed [7]. For the adjuvant setting, the APHINITY study demonstrated a modest but clinically relevant invasive disease-free survival (DFS) gain from adding pertuzumab to standard trastuzumab‑based adjuvant therapy, especially in node‑positive patients, with low cardiac toxicity [10].

Despite evidence supporting the addition of pertuzumab, variability remains in its overall efficacy and safety profile across different clinical settings (neoadjuvant/adjuvant versus advanced/metastatic), highlighting the need for clearer stratification. In addition, the recent publication of long-term overall survival data from pivotal trials such as the third interim analysis of APHINITY and the final analysis of PEONY warrants a comprehensive re-evaluation [7, 10]. Therefore, we conducted an updated systematic review and meta-analysis to provide a more precise assessment of the efficacy and safety of pertuzumab-based regimens across different settings of HER2-positive breast cancer.

## Methods

This systematic review and meta-analysis were conducted in accordance with the Cochrane Handbook for Systematic Reviews of Interventions [11] and are reported following the Preferred Reporting Items for Systematic Reviews and Meta-Analyses (PRISMA) 2020 statement [12]. The registration number for this systematic review on PROSPERO is CRD420261373120.

### Eligibility criteria

Studies were selected according to the PICOS framework:

1. **Population**: Adults (≥ 18 years) with pathologically confirmed HER2-positive breast cancer, regardless of treatment setting (neoadjuvant, adjuvant, locally advanced, or metastatic).
2. **Intervention**: Any treatment regimen including pertuzumab in combination with trastuzumab or chemotherapy.
3. **Comparator**: Regimens without pertuzumab, including placebo, trastuzumab-based therapy alone, or standard chemotherapy and/or endocrine therapy.
4. **Efficacy outcomes**: progression-free survival (PFS), invasive disease-free survival (IDFS), and overall survival (OS).
5. **Safety outcomes**: Incidence of all adverse events (AEs) and grade ≥ 3 AEs.
6. **Study design**: Phase II or III randomized controlled trials (RCTs), including parallel-group, blinded, or open-label designs.

Non-randomized studies, single-arm trials, reviews, meta-analyses, conference abstracts without full-text availability, and studies with insufficient outcome data were excluded.

### Literature search

A comprehensive literature search was conducted from database inception to January 2026 across the following electronic databases: PubMed, Scopus, Web of Science, and Cochrane Library. The search strategy incorporated terms related to Breast Cancer AND HER2-positive AND Pertuzumab. A detailed search string per database is provided in Supplementary Table 1.

No restrictions on language or publication date were imposed. Additional studies were identified through manual screening of reference lists from included articles and relevant reviews.

### Study selection process

All records were screened using Rayyan Software [13]. Screening began with titles and abstracts, followed by full-text assessment. Two reviewers independently conducted screening at both stages, with disagreements resolved through consultation with a third reviewer. The selection process was documented using a PRISMA flow diagram.

### Data extraction

Data were independently extracted by two reviewers using a piloted, standardized Microsoft Excel form. The extracted data included:

1. **Study characteristics**: study ID, country, sample size, follow-up duration, and main conclusions.
2. **Participant characteristics**: age, sex, disease stage, hormone receptor status (estrogen receptor [ER] and progesterone receptor [PR]), and visceral disease involvement whenever available.
3. **Intervention and comparator details**: Drug regimens and duration of treatment.
4. **Outcomes**: Hazard ratios (HRs) with 95% confidence intervals (CIs) for time-to-event outcomes (PFS, IDFS, OS) and risk ratios (RRs) with 95% CIs for dichotomous outcomes (AEs).

### Quality assessment

Risk of bias was independently assessed by two reviewers using the Cochrane Risk of Bias 2 (RoB 2) tool in a predesigned Excel sheet [14]. Studies were classified as:

- **Low risk of bias**: Low risk across all domains.
- **Some concerns**: At least one domain with some concerns, but no high-risk domains.
- **High risk of bias**: At least one high-risk domain or multiple domains with some concerns that reduce confidence in the results.

### Certainty of evidence assessment

The certainty of evidence was evaluated using the Grading of Recommendations, Assessment, Development, and Evaluation (GRADE) approach [15]. GRADE considers five domains: risk of bias, indirectness of evidence, inconsistency (heterogeneity), imprecision in effect estimates, and other considerations (including publication, confounding, etc.). The final rating of certainty is categorized as high, moderate, low, or very low.

### Data synthesis and statistical analysis

Data synthesis was performed using R [16]. Outcomes reported in at least two studies were included in the meta-analysis. Efficacy outcomes were analyzed within their clinically appropriate treatment setting to avoid pooling incompatible endpoints across disease stages: progression-free survival (PFS) was analyzed exclusively in the advanced/metastatic setting, whereas overall survival (OS), which is a universally applicable endpoint, was analyzed to assess treatment effect modifications, with formal stratification by treatment setting (neoadjuvant/adjuvant vs. advanced/metastatic) and a test for subgroup interaction, without presentation of a single pooled cross-setting estimate. Safety outcomes were pooled across all treatment settings, as the incremental toxicity of pertuzumab is independent of disease stage, with each trial comparing an identical backbone regimen with versus without pertuzumab. For time-to-event outcomes, pooled hazard ratios (HRs) with 95% CIs were calculated by the inverse-variance method. For dichotomous outcomes, pooled risk ratios (RRs) with 95% CIs were computed. A random-effects model (restricted maximum-likelihood (REML) variance estimation method) was applied due to anticipated clinical heterogeneity. Statistical heterogeneity was assessed using the Chi² test, the I² statistic and the 95% prediction interval (PI; where k ≥ 4). I² values of 0–40% indicated minimal heterogeneity, 30–60% indicated moderate heterogeneity, 50–90% indicated substantial heterogeneity, and 75–100% indicated considerable heterogeneity. A p-value < 0.10 for the Chi² test was considered statistically significant. Pre-specified subgroup analyses (within studies) were conducted to explore sources of heterogeneity, including hormone receptor status (ER/PR positive vs. negative), HER2 immunohistochemistry (IHC) expression, and age group (≤ 65 vs. >65 years). Sensitivity analyses were performed for main outcomes and heterogeneous adverse events outcomes by sequentially excluding one study at a time to assess the robustness of the findings.

### Individual patient data reconstruction and meta-analysis

For overall survival, individual patient-level, time-to-event data (IPD) were reconstructed from the published Kaplan-Meier curves of all five trials meeting protocol eligibility (Xu 2022/PUFFIN, Perez 2016/MARIANNE, Urruticoechea 2017/PHEREXA, Yamato 2022/PRECIOUS, Swain 2020/CLEOPATRA). For progression-free survival, the same procedure was applied to four of these five trials; Perez 2016/MARIANNE was excluded because it reports only an aggregate PFS hazard ratio and publishes no usable PFS curve.

Kaplan-Meier curves for each trial arm were digitized by calibrated vision-based coordinate extraction against each figure’s axes and printed numbers-at-risk table. Curves reported on a proportion scale (0.0–1.0; Urruticoechea 2017, Yamato 2022) were rescaled to the percentage survival scale before reconstruction; no curve required conversion from a cumulative-incidence scale, as all panels were already plotted as survival curves. Extracted coordinates and numbers-at-risk were processed with the IPDfromKM package, implementing the algorithm of Guyot et al., to reconstruct individual time-and-event-status pairs for each trial arm. Reconstruction accuracy was validated against the source curves using root-mean-square error (RMSE ≤ 0.05), mean absolute error (MAE ≤ 0.02), maximum absolute error (≤ 0.05), and Mann-Whitney testing of reconstructed versus digitized survival probabilities, alongside cross-checks of reconstructed event counts and curve endpoints against each trial’s published figures; all reconstructed arms met the pre-specified thresholds. To preserve a consistent treatment-effect direction across trials irrespective of each trial’s own reporting convention, the pertuzumab-containing arm was coded as the reference category [1] and the non-pertuzumab comparator arm as 0 in every trial and both endpoints during reconstruction.

The validated pseudo-IPDs were used in two complementary ways, consistent with standard two-stage IPD meta-analytic practice:

1. *Pooled treatment effect.* A Cox proportional-hazards model was fitted to each trial’s reconstructed IPD to obtain a trial-level log-hazard ratio for the pertuzumab-containing regimen versus control. These were pooled separately for OS and PFS using the generic inverse-variance, random-effects method (REML, Hartung-Knapp), generating pooled HR for each endpoint.
2. *Pooled survival curve.* Survival probability for each arm was estimated from the reconstructed IPD at monthly intervals. At each time point, the contributing trials’ arm-specific survival estimates were combined across studies using random-effects meta-analysis on the complementary log-log scale, separately for the pertuzumab-containing and control arms, generating a pooled survival curve per arm with a pointwise 95% confidence band. Because follow-up duration varied widely across trials (36–120 months), the pooled curve is based on progressively fewer trials as follow-up extends, and beyond each endpoint’s last multi-trial time point (~ 65 months for OS, ~ 53 months for PFS) it reflects Swain 2020/CLEOPATRA alone; this transition is indicated on each figure. For this repeated, time-point-wise pooling procedure specifically, the classical (non-HKSJ) random-effects confidence interval was used in place of HKSJ: with as few as one to two trials contributing at later time points, repeated application of the Hartung-Knapp adjustment is a recognized degenerate case and was avoided; the HKSJ adjustment was retained for the pre-specified pooled hazard ratios described above.

Reconstructed vs. published pooled HR: Throughout, the originally reported pooled hazard ratios from the source trials/review remain the primary estimates of treatment effect; the pooled HR generated from the reconstructed IPD is reported as an internal validation and sensitivity measure, not as a replacement for it. This reflects an inherent limitation of curve-based reconstruction rather than of the pooling method itself: because reconstruction relies on digitized, calibration-dependent coordinates and numbers-at-risk rather than the true patient-level records, and because the resulting Cox models are necessarily unstratified (only time, event status, and arm survive digitization, whereas several published trial-level HRs are adjusted or stratified for randomization factors), the reconstructed HR can drift from the published estimate even when the underlying curve fit meets pre-specified accuracy thresholds. This was evident in the present reconstruction, where two trials with coarser numbers-at-risk grids (Perez 2016/MARIANNE, Urruticoechea 2017/PHEREXA) showed the largest such deviations on validation, while trials with finer-grained risk tables reconstructed within a few percent of their published HR. The PFS reconstruction is a further case in point, since it pools only four of the five trials in the published analysis. The reconstructed pooled HR is therefore presented alongside, and cross-checked against, the published pooled HR, with any material discrepancy attributed to reconstruction rather than to a true difference in treatment effect.

## Results

### Studies included

**Fig. 1:**
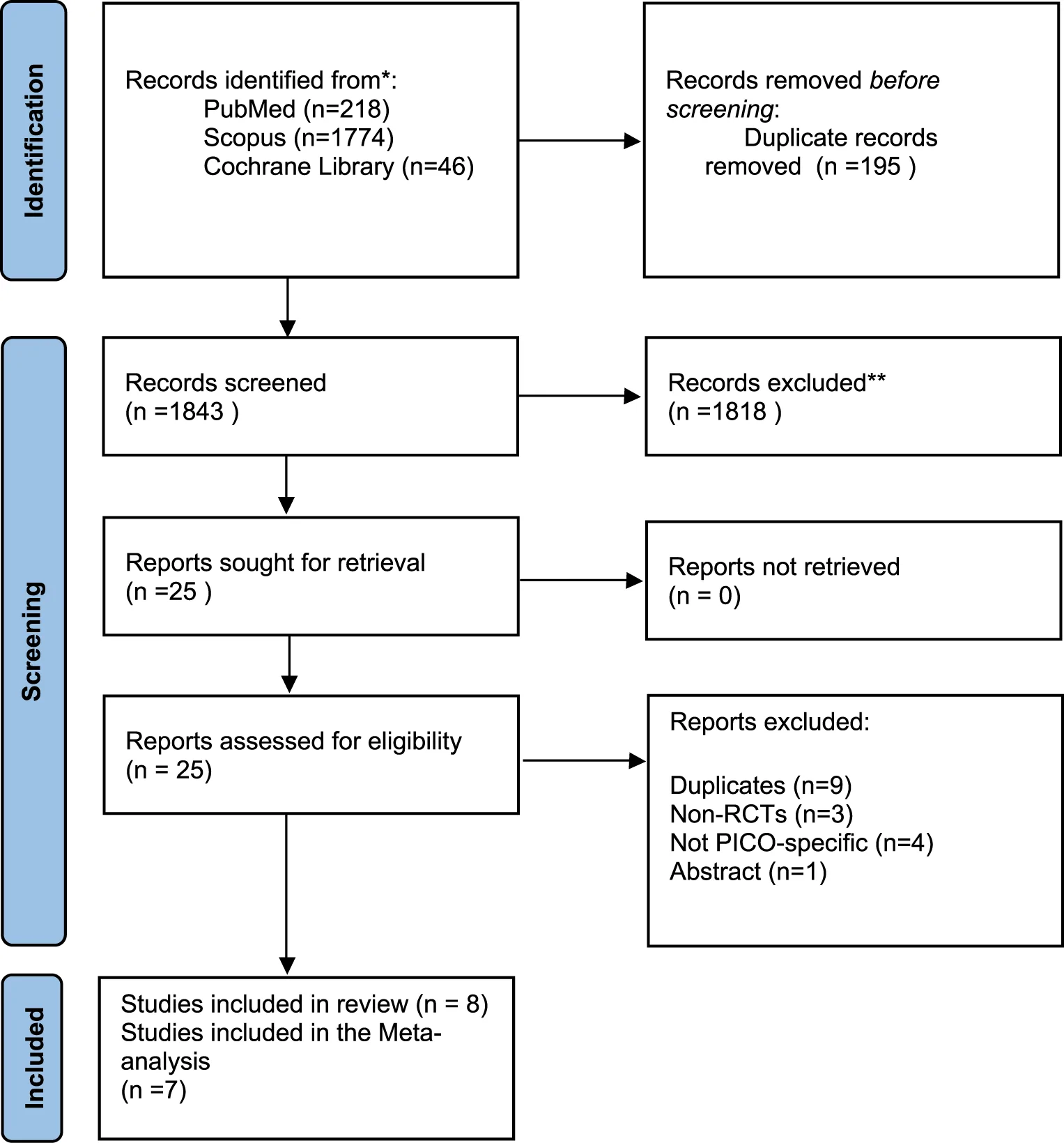
PRISMA flowchart for the study selection process

The database search identified 2,038 records. After removing 195 duplicates, 1,843 unique records underwent title and abstract screening. Of these, 25 studies were assessed for full-text eligibility, and 8 studies met the inclusion criteria and were included in the final analysis, as shown in Fig. 1.

### Study characteristics

We included 8 RCTs with 8367 patients [7, 8, 10, 17–21]. Most of the trials were multicentric, except for two trials set in China and Japan [17, 18, 22]. The intervention arm included Pertuzumab, trastuzumab, and another chemotherapy in most trials, except for one trial that included Pertuzumab and trastuzumab only. All study characteristics and baseline population characteristics are included in Tables 1 and 2.

**Table 1:** Study characteristics of included studies

Study ID	Trial name	Country	Sample Size	Treatment Strategy	Treatment arm	Conclusion
HUANG 2024	PEONY	Multicentric	329	Neoadjuvant therapy, followed by adjuvant (early)	Trastuzumab plus Pertuzumab plus Chemotherapy	Combinations of trastuzumab and pertuzumab have improved patient outcomes over the years and are the standard of care in HER2-positive breast cancer. Consistently, the EFS analysis in PEONY demonstrated a clinically meaningful long-term benefit with this combination.
Loibl 2024	APHINITY	Multicentric	4804	Adjuvant (early)	Pertuzumab, trastuzumab, and chemotherapy	Overall, iDFS improvement after follow-up; no late cardiac toxicity with pertuzumab; pertuzumab recommended for treating HER2-positive BC
XU 2022	PUFFIN	China	243	Metastatic / locally advanced	Pertuzumab + Trastuzumab + Docetaxel	PUFFIN adds to the totality of data with pertuzumab in previously untreated HER2-positive locally recurrent or metastatic breast cancer and supports the favorable benefit-risk profile of pertuzumab in Chinese patients
Yamato 2022	PRECIOUS	Japan	219	Metastatic / locally advanced	Pertuzumab, trastuzumab, and chemotherapy	PFS improved by adding pertuzumab, better OS, and duration of response
Swain 2020	CLEOPATRA	Multicentric	808	Metastatic, not CNS metastasis	Pertuzumab plus trastuzumab plus docetaxel	Increase in the overall survival rate
Urruticoechea 2017	PHEREXA	Multicentric	452	Metastatic, not CNS metastasis	Pertuzumab + trastuzumab + capecitabine	The addition of pertuzumab didn’t significantly improve IRF PFS, 8 months increase in median OS
Gianni 2016	NeoSphere	Multicentric	417	Early (neoadjuvant/ locally advanced)	Pertuzumab + Trastuzumab + Docetaxel	Neoadjuvant pertuzumab + trastuzumab + docetaxel trends toward improved long-term outcomes compared with trastuzumab + docetaxel; results support a benefit, particularly in hormone receptor-negative disease; good long-term safety profile.
Perez 2016	MARIANNE	Multicentric	1095	Locally advanced	trastuzumab + pertuzumab	T-DM1 versus T-DM1 plus pertuzumab showed no notable differences in PFS

**Table 2:** Baseline characteristics of included studies

Trial	Arms	Total number	Age (Mean (SD))	PR/ER status (+ ve positive)(%)	PR/ER status (-ve negative)	Visceral disease involvement (%)
HUANG 2024	Pertuzumab Arm	219	48.5 (9.5)	114 (52.1%)	105 (47.9%)	104 (47.5%)
	Placebo arm	110	49.3 (9.5)	55 (50%)	55 (50%)	55 (50%)
Loibl 2024	Pertuzumab, trastuzumab and chemotherapy	2,400	NA	1,536 (64.0%)	864 (36.0%)	NA
	Placebo, trastuzumab and chemotherapy	2,404	NA	1,546 (64.3%)	858 (35.7%)	NA
XU 2022	Pertuzumab arm (pertuzumab + trastuzumab + Docetaxel)	122	51.8 (11.5)	53 (43.4%)	69 (56.6%)	88 (72.1%)
	Placebo arm (Placebo + Trastuzumab + Docetaxel)	121	53.3 (12.1)	48 (39.7%)	73 (60.3%)	86 (71.1%)
Yamato 2022	Pertuzumab, trastuzumab, and chemotherapy (Intervention)	108	55.5 (10.69)	58 (53.7%)	50 (46.3%)	73 (67.6%)
	Trastuzumab and chemotherapy	109	58.75 (10.08)	64 (58.7%)	45 (41.3%)	75 (68.8%)
Swain 2020	Pertuzumab, trastuzumab, and docetaxel	334	NA	NA	NA	263 (78.74%)
	Placebo, trastuzumab, and docetaxel	339	NA	NA	NA	271 (79.9%)
Urruticoechea 2017	Trastuzumab, capecitabine (control)	224	NA	123 (55.0%)	98 (44.0%)	146 (65.5%)
	Pertuzumab, trastuzumab, capecitabine (intervention)	228	NA	126 (55.0%)	99 (43.0%)	148 (65.5%)
Gianni 2016	Group A (Trastuzumab + Docetaxel)	107	49.6 (10.05)	50 (47%)	57 (53%)	N/A
	Group B (Pertuzumab + Trastuzumab + Docetaxel)	107	50.9 (8.94)	50 (47%)	57 (53%)	N/A
	Group C (Pertuzumab + Trastuzumab)	107	50 (9.5)	51 (48%)	55 (52%)	N/A
	Group D (Pertuzumab + Docetaxel)	96	50 (9.5)	46 (48%)	50 (52%)	N/A
Perez 2016	Trastuzumab, taxane	365	55 (11.27)	207 (56.7%)	154 (42.2%)	241 (66.0%)
	Trastuzumab + pertuzumab	363	54.2 (10.08)	198 (54.5%)	156 (43.0%)	259 (71.3%)

### Quality assessment

**Fig. 2:**
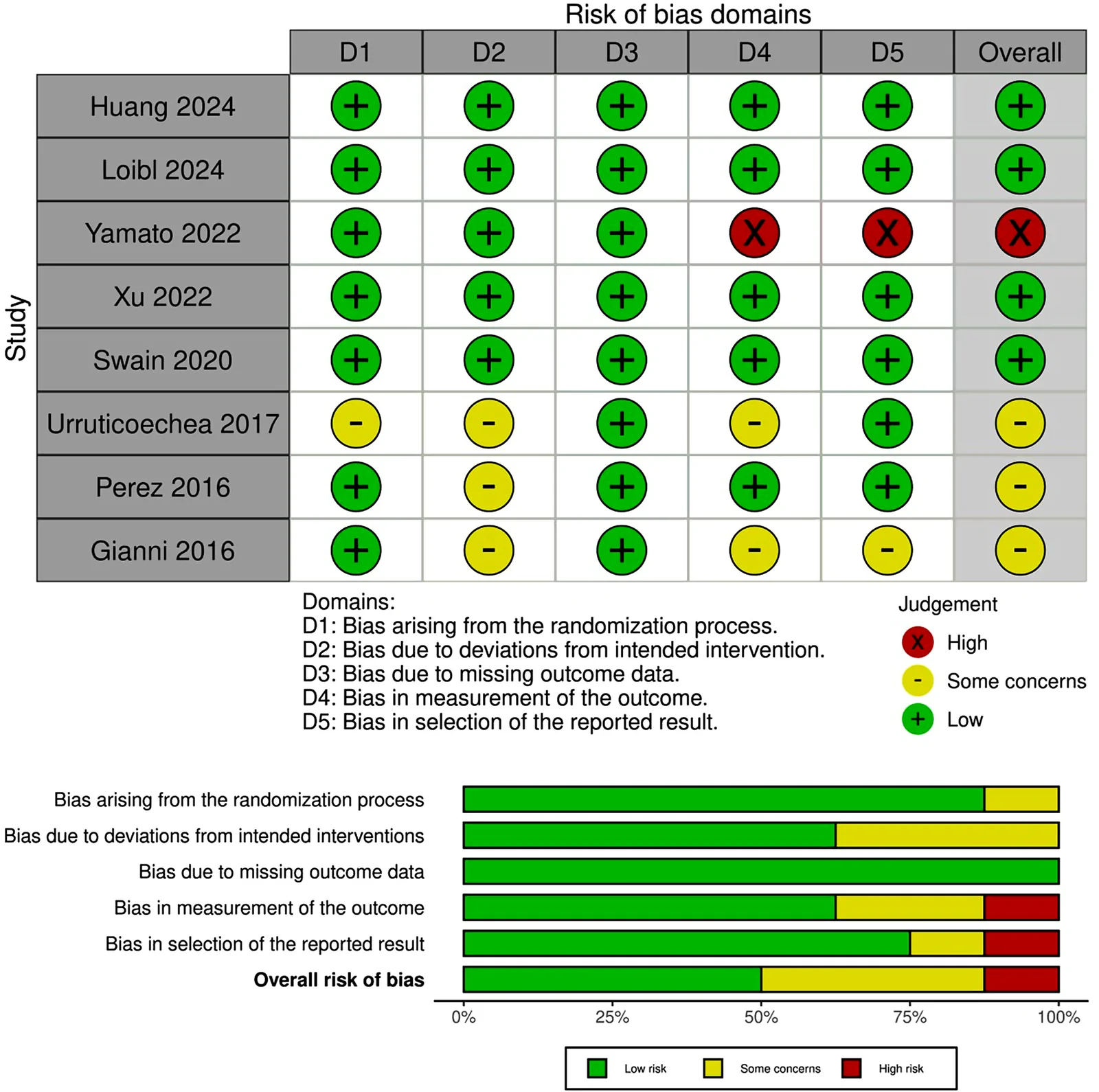
Risk of bias graph and summary using the ROB 2.0 tool

Four studies (Huang 2024, Loibl 2024, Xu 2022, and Swain 2020) were judged to have an overall low risk of bias across all domains. Three studies (Urruticoechea 2017, Perez 2016, and Gianni 2016) were rated with some concerns, primarily due to deviations from intended interventions (D2), measurement of outcomes (D4), and selection of reported results (D5). One study (Yamato 2022) was judged to have a high overall risk of bias. The risk-of-bias summary and bias graph for the included studies are shown in Fig. 2.

### Efficacy outcomes

#### Overall survival

**Fig. 3:**
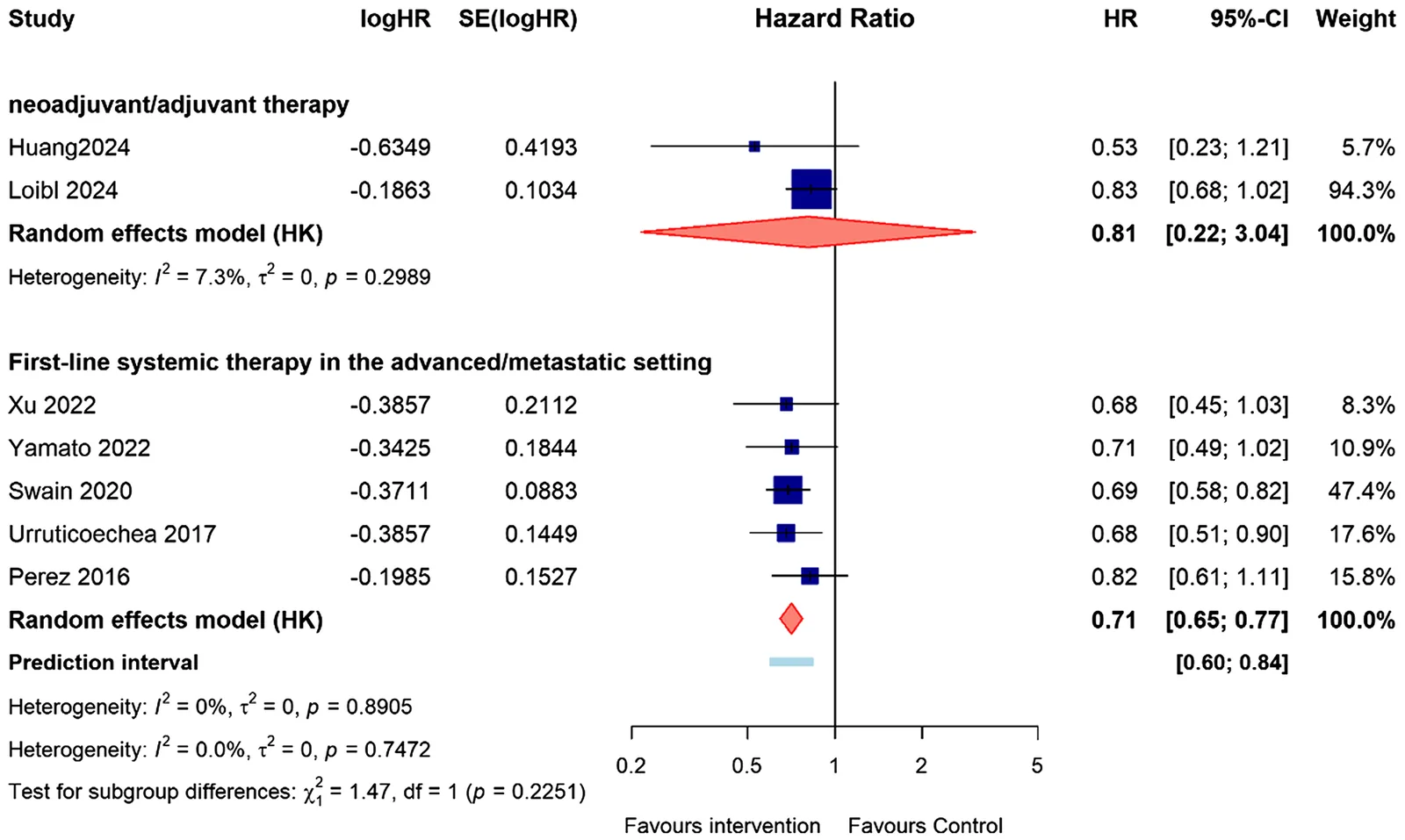
Forest plot of overall survival, stratified by treatment setting (neoadjuvant/adjuvant vs. advanced/metastatic); no pooled overall estimate is presented

**Fig. 4:**
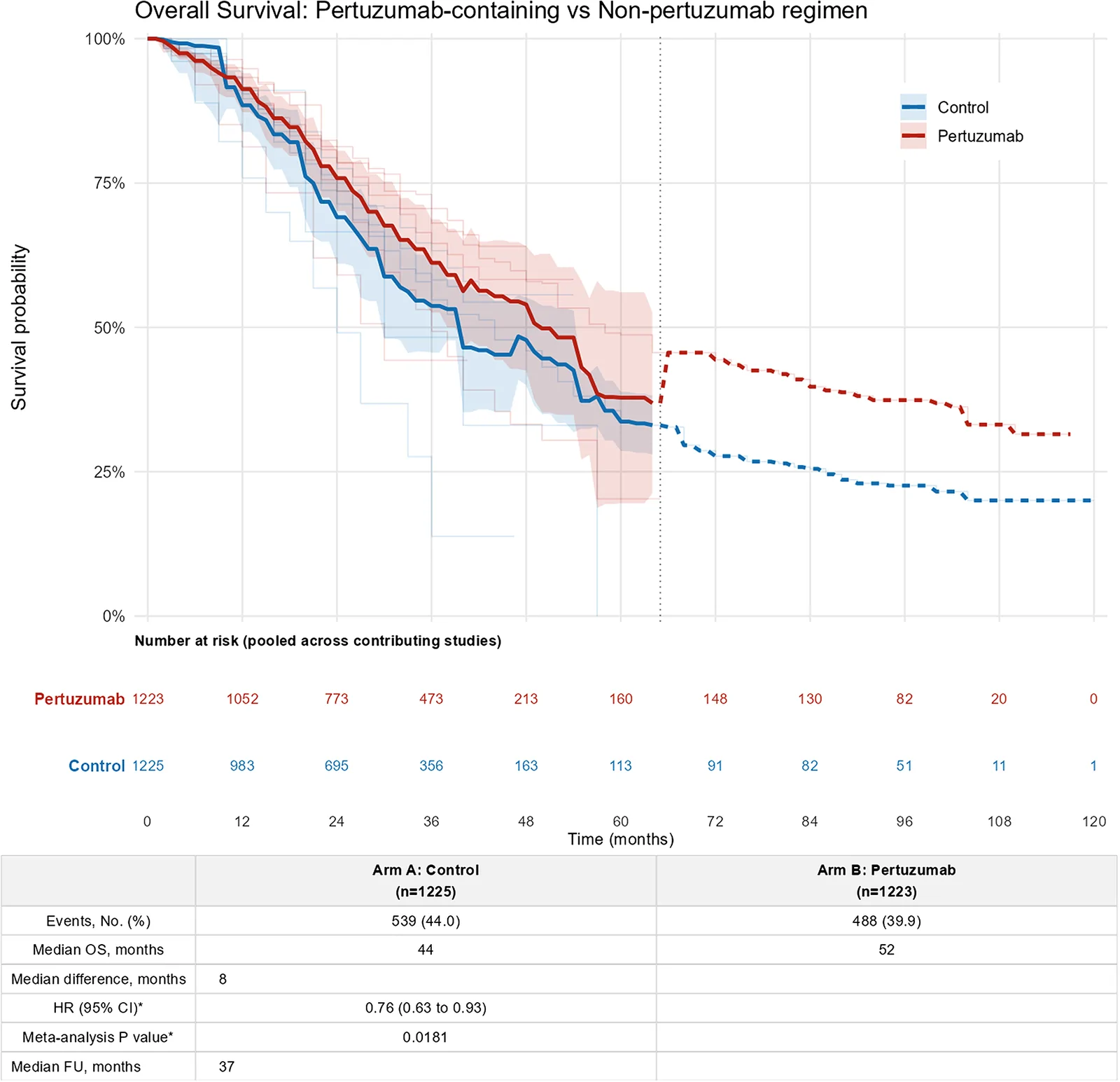
Kaplan-Meier curve for Overall Survival. Random-effects pooled reconstructed IPD, 5 trials (complete OS pool). Thin lines = individual-trial KM curves; bold lines = pooled estimate ± 95% CI. Dashed segment (> 65 months) = Swain 2020/CLEOPATRA alone, not pooled. HR/CI/P-value = random-effects estimate; events and median OS are crude pooled-patient counts and may not track the HR’s direction

Seven RCTs provided overall survival (OS) data (Figs. 3 and 4). In the neoadjuvant or adjuvant setting (2 trials), the pooled HR was 0.81 (95% CI 0.22–3.04), with no heterogeneity detected among these trials (I² = 7.3%; *p* = 0.2990). In the advanced or metastatic setting (5 trials), the benefit was numerically greater, with an HR of 0.71 (95% CI 0.65–0.77; PI = 0.60–0.84; I² = 0%). There was no significant difference between the two settings (p *=* 0.22); the unequal distribution (k = 2 vs. k = 5) constrains the precision of its estimate. The certainty of evidence for overall survival across all settings was rated as moderate, primarily downgraded for risk of bias in metastatic trials and for imprecision in early-disease trials due to limited sample size and wide confidence intervals in the neoadjuvant/adjuvant subgroup (Supplementary Tables 2, 3).

#### Progression-free survival

**Fig. 5:**
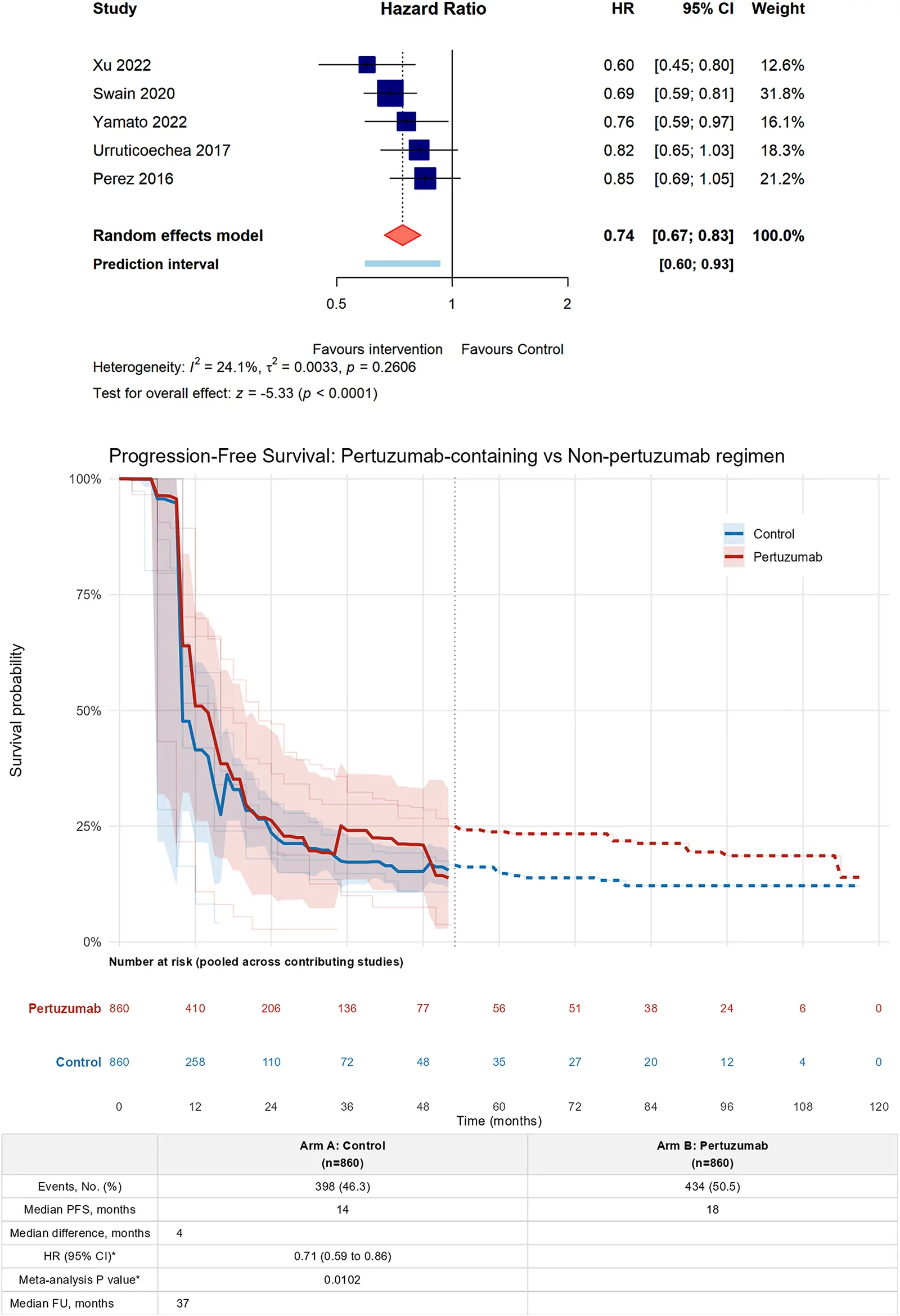
Forest plot of progression-free survival in the advanced/metastatic setting. **A**. Kaplan-Meier curve for PFS Survival. Random-effects pooled reconstructed IPD, 4 of 5 trials (Perez 2016 excluded, no usable PFS curve, 22.1% of published weight). Thin lines = individual-trial KM curves; bold lines = pooled estimate ± 95% CI. Dashed segment (> 53 months) = Swain 2020/CLEOPATRA alone, not pooled. HR/CI/P-value = random-effects estimate; events and median PFS are crude pooled-patient counts and may not track the HR’s direction

Five RCTs contributed to the primary analysis of progression-free survival (PFS), enrolling patients across both metastatic and locally advanced settings (Fig. 5, 5.A). Pertuzumab-containing regimens conferred a significant 26% reduction in the hazard of disease progression compared with control (HR 0.74; 95% CI 0.67–0.83; *p* < 0.0001). There was no significant heterogeneity observed (I² = 24.1%; τ² = 0.0033; *p* = 0.26), with a prediction interval that did not cross the null (PI = 0.60–0.93). The certainty of evidence for progression-free survival was moderate, with downgrading due to concerns regarding the risk of bias in the included trials (Supplementary Table 4).

#### Pre-specified subgroup analyses

**Fig. 6:**
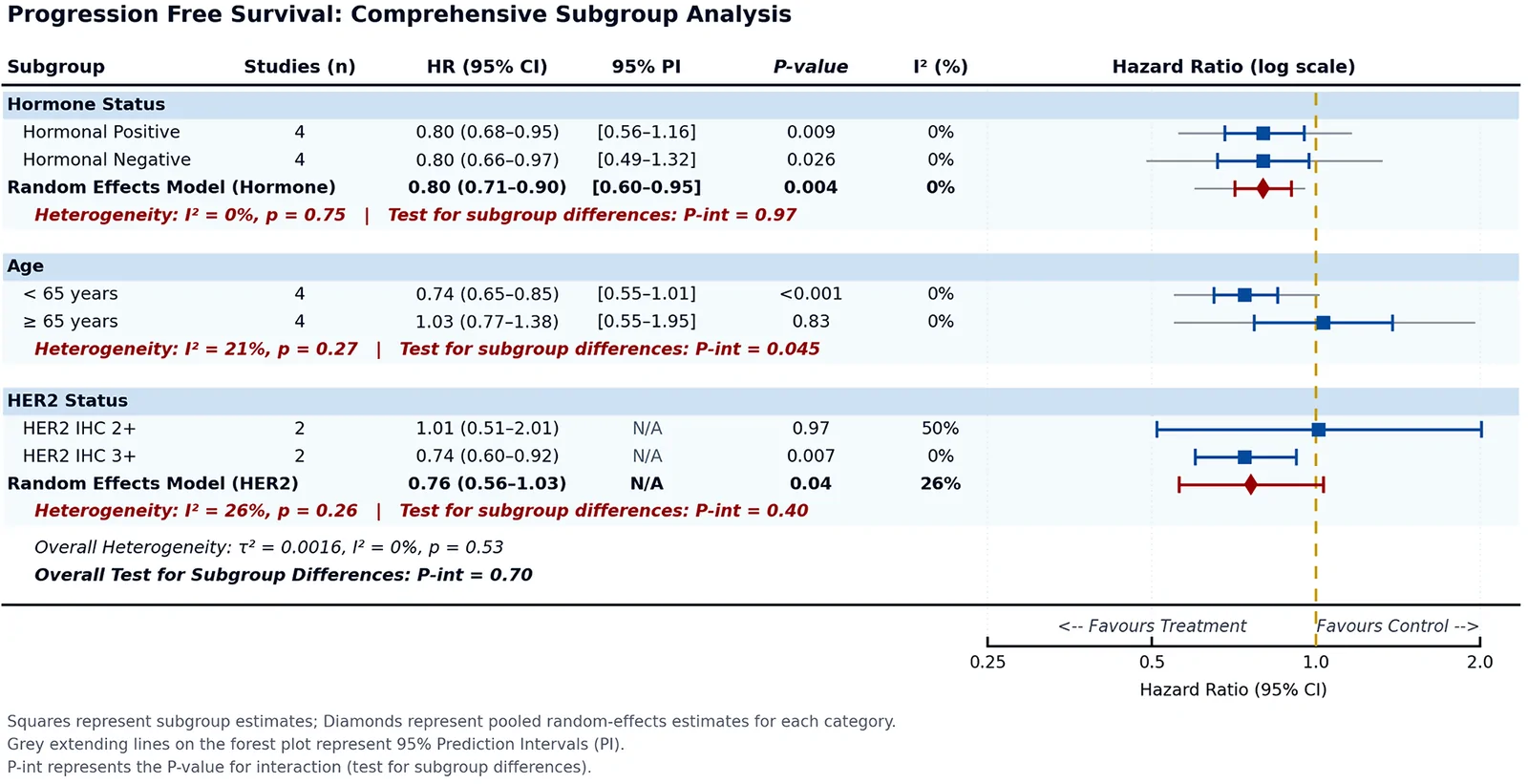
Progression-free survival subgroup by hormone receptor status, age (< 65 vs. ≥65 years), and HER2 IHC expression (2 + vs. 3+) in the advanced/metastatic setting. “Studies (n)” denotes the number of within-study subgroup strata contributing to each comparison, not unique trials; individual trials report multiple strata and therefore appear in more than one row

A comprehensive subgroup analysis of PFS across three clinically relevant patient characteristics: hormone receptor (HR) status, age, and HER2 IHC expression was summarized (Fig. 6).

Hormone receptor status did not modify the effect of pertuzumab on PFS. Both HR-positive (4 trials; HR 0.80, 95% CI 0.68–0.95; *p* = 0.009; I² = 0%) and HR-negative patients (4 trials; HR 0.80, 95% CI 0.66–0.97; *p* = 0.026; I² = 0%) derived significant and equivalent PFS benefit, with no evidence of interaction (P-int = 0.97). The pooled estimate across all eight hormone-stratified studies was HR 0.80 (95% CI 0.71–0.90; *p* = 0.004; I² = 0%), with a prediction interval of 0.60–0.95 (Supplementary Figure 1).

In the age-stratified analysis, a significant PFS benefit was demonstrated in patients younger than 65 years (HR 0.74; 95% CI 0.65–0.85; *p* < 0.0001; I² = 0%), whereas in those aged 65 years or older, there was no significant difference between the pertuzumab and control (HR 1.03; 95% CI 0.77–1.38; *p* = 0.83; I² = 0%). The test for subgroup interaction was significant (P-int = 0.04) (Supplementary Figure 2).

Among four trials stratified by HER2 IHC expression, patients with IHC 3 + tumours demonstrated a significant PFS benefit (HR 0.74; 95% CI 0.60–0.92; *p* = 0.007; I² = 0%). In contrast, those with IHC 2 + tumours showed no significant effect (HR 1.01; 95% CI 0.51–2.01; *p* = 0.97; I² = 50%). However, the test for subgroup differences was not statistically significant (P-int = 0.40), and only two trials contributed to each stratum, substantially limiting power to detect a true interaction (Supplementary Figure 3).

### Safety outcomes

**Fig. 7:**
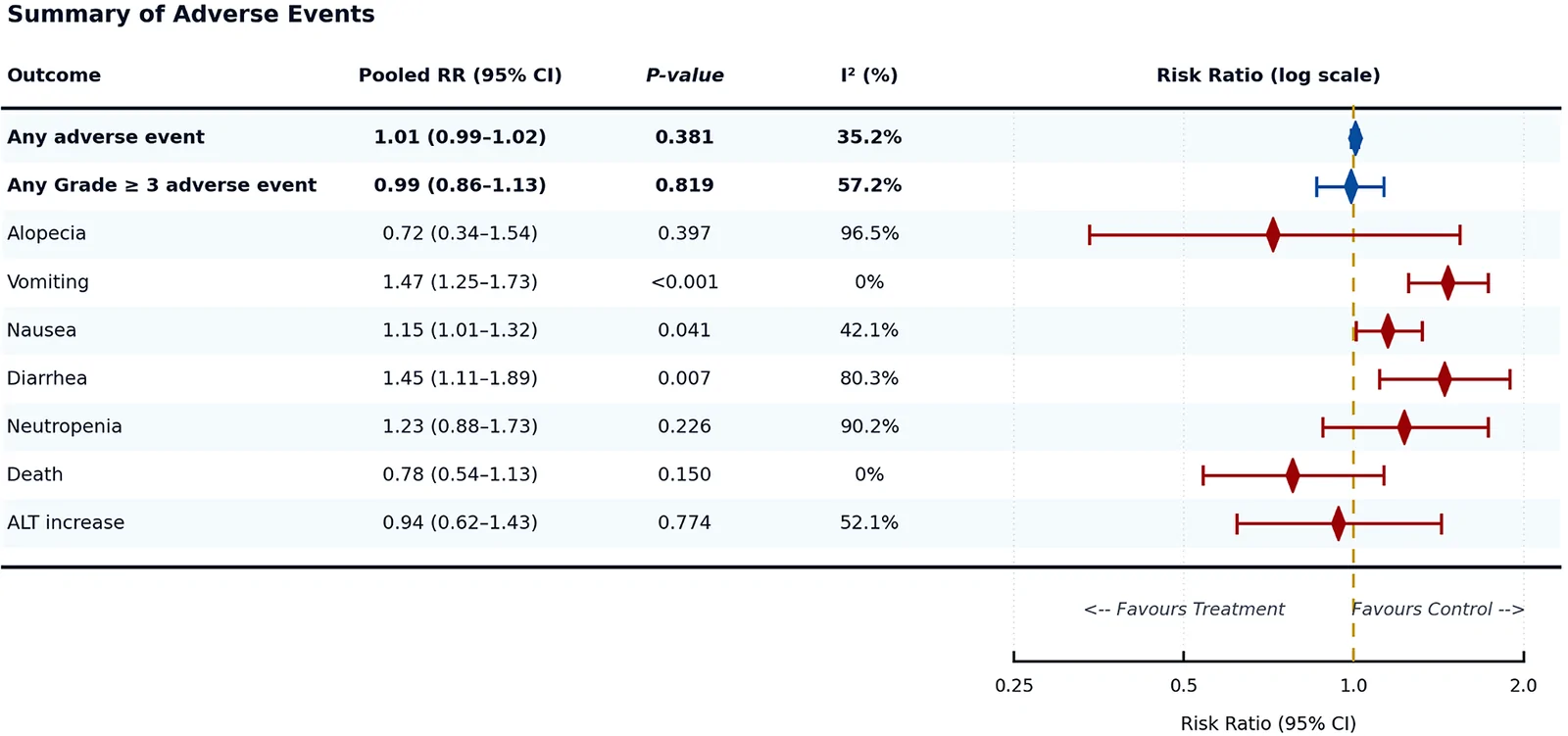
Summary forest plot of pooled risk ratios for all adverse event outcomes. Pooled estimates are shown with classical random-effects 95% CIs, which differ from the Hartung–Knapp–Sidik–Jonkman (HKSJ)-adjusted 95% CIs and *p*-values reported in the text

All safety outcomes were summarised in (Fig. 7).

#### Overall and grade ≥ 3 adverse events

Among six trials reporting overall adverse event (AE) incidence (experimental arm: 1136 patients; control arm: 1,026 patients), the addition of pertuzumab did not significantly increase the risk of experiencing any adverse event (RR 1.01; 95% CI 0.99–1.02; I² = 35.2%; PI 0.98–1.03) (Supplementary Figure 4).

The incidence of grade ≥ 3 adverse events (7 trials; experimental: 3500; control: 3431) was similarly not significantly increased by pertuzumab (RR 0.99; 95% CI 0.86–1.13) (Supplementary Figure 5). However, the substantial heterogeneity for this outcome (I² = 57.2%; τ² = 0.0085; *p* = 0.029) and a wide prediction interval (0.76–1.28) that extends above unity indicate significant variability in grade ≥ 3 toxicity profiles across trials.

#### Specific adverse events and absolute risk estimates

Analysis of individual AE categories identified two gastrointestinal toxicities occurring at significantly higher rates in the pertuzumab arm (Supplementary Figure 6).

Vomiting was the most markedly elevated toxicity (5 trials; RR 1.47; 95% CI 1.29–1.69; *p* = 0.0013; I² = 0%). Against an observed control-arm vomiting rate of 20.0% (181 events/906 patients), this corresponds to an absolute risk increase of 9.4% points, a number needed to harm (NNH) of 11, and an estimated 94 excess vomiting events per 1,000 patients treated with pertuzumab. There was no heterogeneity observed (I² = 0%; *p* = 0.85).

Diarrhoea was significantly more frequent with pertuzumab (6 trials; RR 1.45; 95% CI 1.02–2.06; *p* = 0.042). Against a control-arm diarrhoea rate of 41.4% (421/1,016), this translates to an absolute risk increase of 18.6% points and an NNH of approximately 5, equivalent to 186 excess diarrheal events per 1,000 treated patients. However, significant heterogeneity was present (I² = 80.3%; *p* = 0.0001).

In contrast, no significant between-arm differences were observed for nausea (RR 1.15; 95% CI 0.99–1.35; *p* = 0.067; I² = 42.1%), alopecia (RR 0.72; 95% CI 0.24–2.12; *p* = 0.44; I² = 96.5%), neutropenia (RR 1.23; 95% CI 0.77–1.97; *p* = 0.30; I² = 90.2%), treatment-related death (RR 0.78; 95% CI 0.54–1.13; *p* = 0.14; I² = 0%), or ALT elevation (RR 0.94; 95% CI 0.45–1.97; *p* = 0.80; I² = 52.1%).

### Sensitivity analyses

Leave-one-out sensitivity analyses across all three primary efficacy outcomes confirmed the robustness of pooled estimates against undue influence from any single trial, a particular concern given the high weight of Loibl 2024 in the OS analysis (94.3%).

For OS, estimates were similarly stable across all five metastatic studies (HR range 0.69–0.73), with prediction intervals persistently below unity (Supplementary Fig. 7). For PFS, sequential omission of each of the five studies yielded HR estimates ranging from 0.72 to 0.77, all remaining statistically significant, with the narrow 0.05-unit range confirming no trial disproportionately drove the conclusion (Supplementary Fig. 8).

For Grade ≥ 3 adverse events, sequential omission of each trial produced pooled risk ratios between 0.95 (95% CI 0.83–1.10; omitting Urruticoechea 2017) and 1.02 (0.88–1.18; omitting Perez 2016). All confidence and prediction intervals crossed unity and residual heterogeneity remained moderate throughout (I² 48.1–62.6%, τ² 0.0044–0.0116). The pooled estimate (RR 0.99, 95% CI 0.86–1.13; 95% PI 0.76–1.28; I² = 57.2%) therefore does not depend on any single trial, and the finding of no excess in high-grade toxicity is robust. (Supplementary Fig. 9)

For alopecia, the pooled estimate was driven almost entirely by Perez 2016. With that trial excluded, the risk ratio moved from 0.72 (95% CI 0.24–2.12) to 1.03 (0.90–1.16) and between-study heterogeneity was abolished (I² 96.5% → 0%, τ² 0.7307 → 0). Omitting any other trial left the estimate essentially unchanged (RR 0.63–0.67) with I² ≈ 97%. The apparent reduction in alopecia is thus attributable to one outlying trial (RR 0.15, 95% CI 0.11–0.21) and should not be interpreted as a treatment effect. (Supplementary Fig. 10)

For neutropenia, leave-one-out estimates ranged from 0.99 (95% CI 0.78–1.27; omitting Perez 2016) to 1.34 (0.76–2.36; omitting Gianni 2016), and every interval included unity, consistent with the pooled result (RR 1.23, 95% CI 0.77–1.97; I² = 90.2%). Perez 2016 accounted for most of the heterogeneity: its exclusion reduced I² from 90.2% to 64.3% and τ² from 0.1551 to 0.0050. The conclusion of no significant difference in neutropenia is stable, although the magnitude of the estimate remains imprecise. (Supplementary Fig. 11).

## Discussion

Our GRADE-assessed meta-analysis provides an overview of the effect of pertuzumab on HER2-positive breast cancer. Our meta-analysis demonstrates a highly significant overall survival (OS) benefit with the addition of pertuzumab to standard therapy in patients with metastatic HER2-positive breast cancer. The observed around 20% relative reduction in the hazard of death represents a clinically meaningful therapeutic gain in both the early and metastatic stages of this population.

OS benefit in each subgroup is consistent with the magnitude of survival improvement reported in key trials, with the metastatic setting representing a particular strength of our analysis given the greater number of available trials and more mature survival data from trials such as CLEOPATRA (*n* = 808 first-line metastatic patients). Particularly in the PRECIOUS study [18], the addition of pertuzumab to trastuzumab and docetaxel in metastatic HER2-positive breast cancer reduced the risk of death by approximately 30%.

At the same time, the APHINITY (*n* = 4,804 operable HER2-positive patients) and PEONY (*n* = 329 Asian patients with early or locally advanced disease) trials demonstrated invasive and event-free survival benefits alongside favorable overall survival trends in the early-stage setting, suggesting that our setting-specific findings lie within the range of the most robust individual trial results [7, 10]. However, the relatively small number of trials in the early stages may limit generalizability in this population.

The superior efficacy of dual HER2 blockade compared with single-agent HER2-targeted therapy may be attributable to its ability to overcome resistance mechanisms. Single-agent HER2 blockade can be undermined by activation of the PI3K/AKT signaling pathway, loss of PTEN function, and cross-talk with other receptor pathways, allowing tumor cells to bypass HER2 inhibition [23]. These resistance mechanisms provide a strong biological rationale for combining HER2-directed antibodies [24]. Pertuzumab complements trastuzumab by inhibiting HER2 dimerization and downstream signaling pathways, resulting in more comprehensive HER2 blockade and potentially overcoming resistance that may limit the effectiveness of single-agent therapy.

In addition, our meta-analysis demonstrates that pertuzumab-containing regimens reduced the hazard of disease progression by 26% (95% CI 0.67–0.83), with no heterogeneity observed. This finding aligns with the improvement in progression-free survival (PFS) observed across the included trials, with landmark studies such as CLEOPATRA [8] contributing to the demonstrated benefit of dual HER2 blockade in the metastatic setting.

Our subgroup analyses indicate that receptor status did not modify the effect of pertuzumab on progression-free survival (PFS). The comparable benefit observed irrespective of estrogen receptor co-expression is clinically informative, suggesting that pertuzumab’s mechanism of HER2 dimerization inhibition functions independently of hormone receptor signaling pathways. In contrast, the test for subgroup interaction by age was statistically significant (P-int = 0.04).

Although there was a trend toward a reduced magnitude of benefit in patients aged ≥ 65 years, the treatment effect in this subgroup was not statistically significant. This apparent attenuation should be interpreted with caution, as the wider confidence intervals in the older subgroup likely reflect fewer available studies and lower event density. As such, the non-significant PFS finding in older patients is more plausibly explained by limited statistical power rather than a true absence of treatment effect.

Importantly, subgroup analyses in RCT meta-analyses are inherently exploratory and require a statistically significant interaction test (P-int) to support claims of effect modification [25]. In the absence of such evidence, observed differences in point estimates across subgroups should not be interpreted as indicating differential efficacy.

For safety outcomes, the pooled risk ratio for overall adverse events was 1.00, with a narrow confidence interval, indicating that the addition of pertuzumab does not meaningfully increase the overall adverse event burden.

The observed heterogeneity in grade ≥ 3 adverse events is most plausibly explained by variation in background chemotherapy regimens (e.g., docetaxel, carboplatin, capecitabine) across the included trials, rather than inconsistency in the effect of pertuzumab itself. A similar explanation likely underlies the variability observed in the incidence of diarrhea.

In contrast, the prediction interval for vomiting lies entirely above unity, suggesting that this increased risk is consistent across study settings and can be anticipated in routine clinical practice [26].

Reassuringly, the near-null risk ratios for treatment-related mortality and ALT elevation, together with the absence of heterogeneity for treatment-related death, provide strong evidence that pertuzumab does not increase the risk of life-threatening toxicity or hepatotoxicity.

Finally, the very high heterogeneity observed for alopecia (I² = 96.5%) and neutropenia (I² = 90.2%) limits the interpretability of these pooled estimates. It likely reflects substantial differences in chemotherapy backbones across trials rather than a direct effect of pertuzumab.

### Limitations and strengths

This study has several limitations. First, disease-free survival (DFS) was excluded from the analysis due to the lack of adjuvant‑only randomized controlled trials evaluating pertuzumab. Second, we excluded the landmark PERTAIN trial [27] evaluating pertuzumab in combination with aromatase inhibitors because its treatment regimen differed from the chemotherapy- and trastuzumab-based regimens evaluated in the other included trials, which could introduce clinical heterogeneity and limit the comparability of the pooled treatment effects. Additionally, methodological restrictions and the potential for bias in some studies, including open-label studies, contributed to residual confounding and resulted in lowered confidence in the evidence according to GRADE evaluation. Factors that affect comparison include inherent differences arising from differing prognostic baselines, accrual rates, and distinct disease stages. These issues pose significant limitations despite using stratified analyses. Moreover, survival analyses in early-stage trials suffer from limited follow-up with small numbers of events, as seen in the case of the PEONY study. Additionally, the fact that treatment crossover was allowed through protocol modification in APHINITY and also done after progression in the PEONY study reduces any detectable differences in overall survival. Finally, the absence of individual patient-level data restricted our ability to perform more specific subgroup analyses by region, comorbidities, or specific biomarker profiles.

For strength, this meta‑analysis incorporates long‑term follow‑up data from key trials such as APHINITY and CLEOPATRA, enabling assessment of mature overall survival rather than only early endpoints. The low heterogeneity (I² = 0.0–26% across outcomes) and narrow prediction intervals entirely below one support the robustness of the observed benefit of pertuzumab across HER2‑positive breast cancer settings.

## Conclusion

The consistent improvements in overall survival and progression-free survival across trials provide strong evidence that pertuzumab-based dual HER2 blockade offers a robust clinical benefit, especially in locally advanced/metastatic stages of the disease.

Importantly, these benefits are achieved without a significant increase in overall toxicity, although predictable adverse events, particularly gastrointestinal effects, require appropriate clinical management.

Overall, this evidence supports the incorporation of pertuzumab into standard treatment strategies. Future research should focus on the use of pertuzumab in early disease settings (neoadjuvant/adjuvant therapy).

## Supplementary Information

Below is the link to the electronic supplementary material.

Supplementary Material 1

## Data Availability

All data generated or analysed during this study are included in this published article and its supplementary information files. The datasets analysed during the current study are available from the corresponding author on reasonable request.
