# Supplementary material for: Pertuzumab in HER2-positive breast cancer: a systematic review and meta-analysis of randomized controlled trials
Source: Breast Cancer Res Treat. 2026 Nov 9;220(1):30. doi: 10.1007/s10549-026-08116-y (PMC13748157; doi:10.1007/s10549-026-08116-y)
Supplement: Supplementary file 1 [file 10549_2026_8116_MOESM1_ESM.docx]

| **Database** | **Search String** |
| --- | --- |
| **PubMed** | ("Breast Neoplasms" OR "breast cancer" OR "breast carcinoma")  AND  ("ErbB-2 receptor" OR "HER2-positive” OR “ERBB2” OR "c-erbB-2")  AND  ("Pertuzumab" OR “Perjeta” OR "rhuMAb 2C4" OR “Omnitarg”) |
| **Scopus** | ("breast cancer" OR "breast carcinoma" OR "breast neoplasm*")  AND  (HER2 OR "HER2-positive" OR ERBB2 OR "c-erbB-2")  AND  (pertuzumab OR Perjeta OR "rhuMAb 2C4" OR Omnitarg) |
| **Web Of Science** | ("breast cancer" OR "breast carcinoma" OR "breast neoplasm")  AND  (HER2 OR "HER2-positive" OR ERBB2 OR "c-erbB-2")  AND  (pertuzumab OR Perjeta OR "rhuMAb 2C4" OR “Omnitarg”) |
| **Cochrane CENTRAL** | ("Breast Neoplasms" OR "breast cancer" OR "breast carcinoma")  AND  ("Receptor, ErbB-2" OR "HER2-positive” OR “ERBB2” OR "c-erbB-2")  AND  ("Pertuzumab" OR “Perjeta” OR "rhuMAb 2C4" OR “Omnitarg”) |

**Supplementary Table 1.** Detailed search strategy per database.

| Outcome | No. of Studies | Study Design | Risk of Bias | Inconsistency | Indirectness | Imprecision | Publication Bias | Certainty |
| --- | --- | --- | --- | --- | --- | --- | --- | --- |
| **Overall Survival** | **5** | **RCT** | **serious** | **Not serious** | **Not serious** | **Not**  **serious** | **None** **detected** | **⨁⨁⨁◯ Moderate** |

**Supplementary Table 2.** GRADE assessment of overall survival for metastatic studies

| Outcome | No. of Studies | Study Design | Risk of Bias | Inconsistency | Indirectness | Imprecision | Publication Bias | Certainty |
| --- | --- | --- | --- | --- | --- | --- | --- | --- |
| **Overall Survival** | **2** | **RCT** | **Not serious** | **Not serious** | **Not serious** | **serious** | **None** **detected** | **⨁⨁⨁◯ Moderate** |

**Supplementary Table 3:** GRADE assessment of overall survival for neoadjuvant/adjuvant studies

| Outcome | No. of Studies | Study Design | Risk of Bias | Inconsistency | Indirectness | Imprecision | Publication Bias | Certainty |
| --- | --- | --- | --- | --- | --- | --- | --- | --- |
| **Progression-free Survival** | **5** | **RCT** | **serious** | **Not serious** | **Not serious** | **Not serious** | **None** **detected** | **⨁⨁⨁◯ Moderate** |

**Supplementary Table 4.** GRADE assessment of progression-free survival

**Supplementary Figure 1.** Forest Plot of Progression-Free Survival Based on Hormonal Status Subgroups.

**Supplementary Figure 2.** Forest Plot of Progression-Free Survival Based on Age Subgroups.

**Supplementary Figure 3.** Forest Plot of Progression-Free Survival Based on HER2 Status Subgroups.

**Supplementary Figure 4.** Forest plot of the incidence of any adverse event, pooled across treatment settings.

**Supplementary Figure 5.** Forest plot of the incidence of grade ≥3 adverse events, pooled across treatment settings.

**Supplementary Figure 6.** Forest plots of individual adverse events, pooled across treatment settings.

**Supplementary Figure 7.** Leave one Sensitivity analysis of Overall Survival for only advanced/metastatic settings.

**Supplementary Figure 8.** Leave-one-out sensitivity analysis of progression-free survival.

**Supplementary Figure 9.** Leave-one-out sensitivity analysis of grade ≥3 adverse events.

**Supplementary Figure 10.** Leave-one-out sensitivity analysis of alopecia adverse events.

**Supplementary Figure 11.** Leave-one-out sensitivity analysis of neutropenia adverse events.
